# Cytomegalovirus Colitis Masquerading as Apple-Core Lesion after Systemic Chemotherapy in a Patient with Relapsed Acute Myeloid Leukemia

**DOI:** 10.1155/2018/5683417

**Published:** 2018-03-20

**Authors:** Jong An, Jason Brownell, Darrell Barker, Theresa Stockinger, Robert Brady, Katherine Cebe, Russell Baur

**Affiliations:** ^1^Department of Hematology and Oncology Service, San Antonio Military Medical Center, Fort Sam Houston, San Antonio, TX 78234, USA; ^2^Department of Internal Medicine, San Antonio Military Medical Center, Fort Sam Houston, San Antonio, TX 78234, USA; ^3^Department of Gastroenterology, San Antonio Military Medical Center, Fort Sam Houston, San Antonio, TX 78234, USA; ^4^Department of Pathology, San Antonio Military Medical Center, Fort Sam Houston, San Antonio, TX 78234, USA

## Abstract

We report the case of a 71-year-old male with relapsed acute myeloid leukemia who developed cytomegalovirus (CMV) colitis presenting as an apple-core lesion during induction chemotherapy. CMV infection occurs rarely during induction chemotherapy for acute myeloid leukemia. CMV infection is usually observed in patients with acquired immune deficiency syndrome (AIDS) and in those on immunosuppressive agents following bone marrow transplant. Although rare, CMV colitis should be considered in patients who are critically ill after systemic chemotherapy as it can cause significant morbidity and mortality.

## 1. Introduction

CMV colitis typically occurs in severely immunocompromised patients with AIDS and in those on immunosuppressive agents after allogenic stem cell transplant or solid organ transplant. Infection in immunocompetent patients is generally asymptomatic or may present as transient symptoms consisting of fever, myalgias, and cervical lymphadenopathy. In recent years, however, there have been a rising number of cases of severe CMV infections in immunocompromised elderly patients with malignancies [1]. Given that CMV seroprevalence rates rise with age, there is a higher risk of reactivation in the setting of chemotherapy among elderly patients leading to significant morbidity and mortality.

This report documents an incidental finding of CMV colitis presenting as an apple-core lesion after chemotherapy in a patient with acute myeloid leukemia.

## 2. Case Presentation

**Figure 1:**
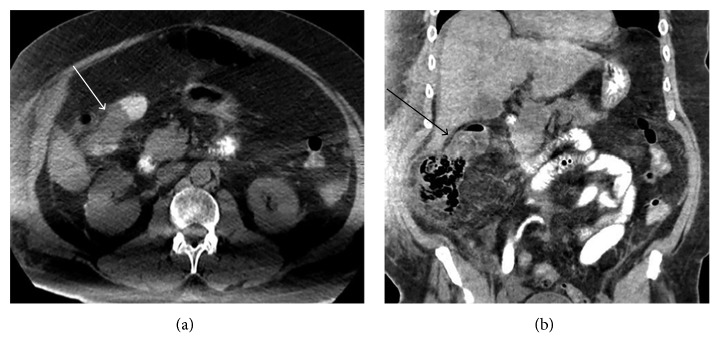
Abdominal computed tomography (CT) demonstrating apple-core mass of the ascending colon.

A 71-year-old man, with a past medical history significant for acute myeloid leukemia, underwent induction with cytarabine and idarubicin followed by consolidation with three cycles of high-dose cytarabine. After one year of achieving remission, he was found to have relapsed leukemia during workup for anemia. He was admitted to the hospital for salvage chemotherapy with cytarabine and mitoxantrone. Ten days after salvage chemotherapy, he developed a neutropenic fever, diarrhea, abdominal pain, and elevated total bilirubin. Abdominal ultrasound showed no signs of biliary obstruction. CT scan of the abdomen and pelvis showed pericecal inflammatory stranding without pneumatosis concerning cecal typhlitis as well as an “apple-core mass” of the ascending colon near the hepatic flexure concerning colon cancer (Figure 1).

**Figure 2:**
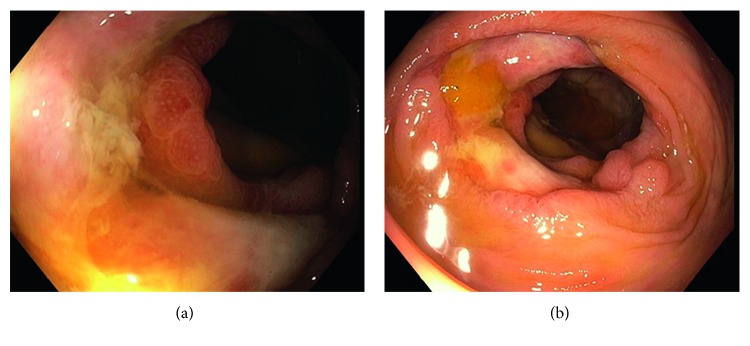
Colonoscopy showing a mass in the proximal transverse colon. Mucosa presents with nodularity, loss of vascular pattern, and diffuse exudates.

Colonoscopy was deferred because he was critically ill with neutropenic enterocolitis. Despite initiation of broad-spectrum antibiotics consisting of vancomycin and piperacillin-tazobactam, his clinical condition deteriorated. He was transferred to the medical intensive care unit with acute respiratory failure requiring assisted ventilation for several days. Blood cultures revealed *Granulicatella adjacens* and transthoracic echocardiogram revealed an aortic valve vegetation consistent with endocarditis requiring 6 weeks treatment with intravenous vancomycin. Additionally, he was noted to have hyperbilirubinemia secondary to hepatic veno-occlusive disease for which he completed a course of defibrotide with eventual normalization of liver enzymes. The patient improved clinically, and subsequent bone marrow biopsy obtained 7 weeks after induction chemotherapy showed no evidence of leukemia. After six weeks of inpatient treatment and subsequent clinical improvement, a colonoscopy was performed to rule out colon cancer given the suspicious apple-core lesion noted on the CT at the hepatic flexure. It revealed diffuse friable changes of the colonic wall extending from the cecum to the hepatic flexure and the transverse colon (Figure 2). Biopsies of the ascending colon, cecum, hepatic flexure, and transverse colon were taken. He was discharged to acute rehabilitation due to deconditioning and a 55 pound weight loss after 7 weeks of inpatient hospitalization.

**Figure 3:**
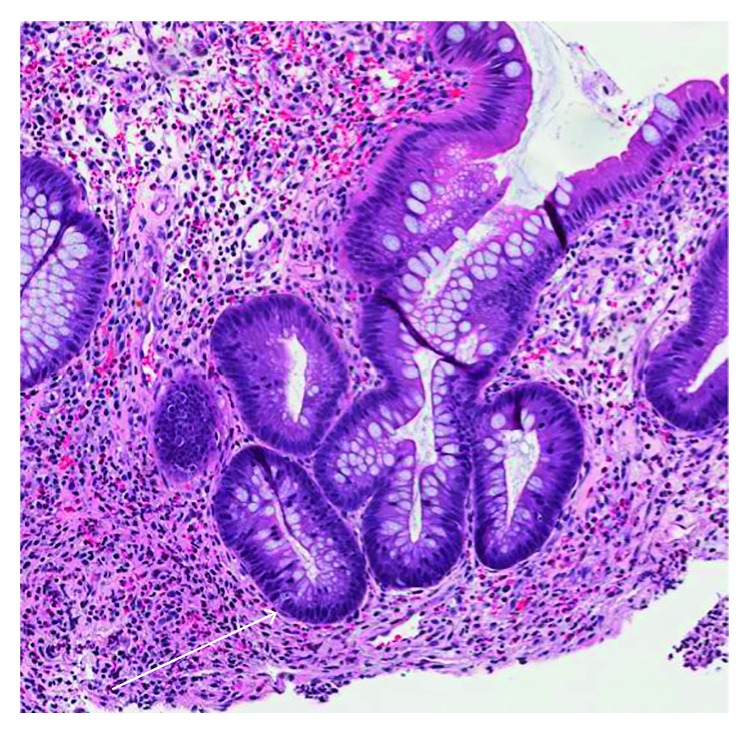
H&E, 126x magnification, showing changes of chronic, active colitis including crypt architectural distortion (branching) and focal active cryptitis (white arrow).

**Figure 4:**
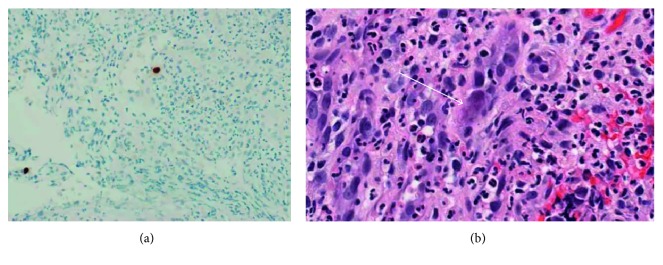
(a) CMV immunohistochemical stain, 200x magnification, highlighting both nuclear and cytoplasmic staining (brown chromogen) within the ulcerated granulation tissue. (b) H&E stain, 400x magnification, showing a CMV-infected endothelial cell with dot-like cytoplasmic inclusions (white arrow).

Final pathology from the cecal biopsy specimens showed chronic active colitis with cryptitis, ulceration, and architectural distortion (Figure 3). CMV immunohistochemical staining was positive for CMV type inclusions compatible with CMV colitis (Figure 4). The biopsies were negative for dysplasia and malignancy. The ascending colon, hepatic flexure mass, and transverse colon mass were negative for CMV inclusions on immunohistochemical stains but showed features consistent with chronic active colitis with cryptitis and architectural distortion.

Our patient remained asymptomatic, without recurrence of diarrhea, fever, or abdominal pain.

## 3. Discussion

We describe a rare case of CMV colitis in a 71-year-old male treated with salvage chemotherapy consisting of cytarabine and mitoxantrone for relapsed acute myeloid leukemia.

CMV remains latent within the host after initial infection and can reactivate later in life. Clinically significant CMV disease is typically seen in profound immunocompromised state such as in AIDS and bone marrow transplant patients. Although CMV status among AIDS and transplant patients is closely monitored with the subsequent initiation of preemptive treatment using antiviral therapy if needed, similar monitoring and treatment plans are not routinely implemented for cancer patients receiving standard chemotherapy. Generally, conventional chemotherapy does not lead to significant alterations in lymphocyte function which is thought to be considered a high risk for the development of CMV disease. However, this paradigm may be changing.

There is increasing incidence of CMV reactivation in patients undergoing conventional chemotherapy. In our literature review, the earliest reported case of CMV colitis after standard chemotherapy was in a patient receiving azacitidine, published in 2011 [2]. A recent study involving 15 cancer patients with 11 head and neck cancers, two lung cancers, one lymphoma, and one rectal cancer showed that all but one patient experienced CMV reactivation during the course of chemotherapy [3]. Ten of the 15 participants had no detectable viral DNA before starting chemotherapy, but the viral load increased in 14 of 15 patients after chemotherapy. The highest CMV viral loads were >100,000 copies/mL in two patients who died. As viral loads increased, patients experienced an increase in symptoms as well as abnormal liver function tests. Another study showed that CMV viremia predicts high mortality rate in cancer patients. The retrospective analysis of cancer patients with CMV viremia showed overall mortality rate of 56% (60/107). This study recommended preemptive antiviral therapy for cancer patients with positive serum CMV PCR results, especially those requiring mechanical ventilation [4]. It is not known in our case whether infection with CMV was present prior to or after the initiation of chemotherapy, as we did not obtain CMV serology.

Because CMV infections commonly occur after allogenic stem cell transplant, these serologic tests are used pretransplant to establish the serostatus which predicts the risk of developing disease, and guides the use of preemptive antiviral therapy. The exact mechanism of the reactivation of CMV is not well established. However, disturbance of the host's immune defenses are thought to play an important role. Male sex, lower body mass index, lymphopenia, hematological malignancy, steroid use, and red blood cell transfusion within one month prior to CMV disease were risk factors for the development of CMV gastrointestinal infection in adult patients with cancer [5]. Our patient possessed several risk factors to include male sex, lymphopenia, and hematological malignancy.

An apple-core lesion of the colon is most frequently associated with obstruction of the colon lumen by colorectal carcinoma but can also be seen in setting of lymphomas, Crohn's disease, ulcerative colitis, ischemic colitis, chlamydia infection, colonic tuberculosis, helminthoma, colonic amoebiasis, villous adenoma, and colonic cytomegalovirus [6]. Thus, tissue biopsy remains the keystone in obtaining the definitive diagnosis.

The gold standard for diagnosing CMV tissue invasive disease is the identification of CMV inclusion or positive CMV immunohistochemistry staining on histopathology [7]. CMV gastrointestinal disease cannot be excluded based on negative blood tests. In a retrospective study of 81 solid organ transplant recipients, 20 cases of biopsy-proven gastrointestinal disease were identified. The sensitivity of PCR for diagnosing CMV gastrointestinal disease was only 85 percent [8]. Plasma CMV DNA was undetectable in three patients with biopsy-proven CMV gastrointestinal disease. Also, gastrointestinal disease may be focal, as seen in this patient. Therefore, multiple biopsies may be needed to confirm the diagnosis of CMV colitis.

Individuals suspected to have CMV colitis should have plasma or whole blood PCR testing since PCR results are often available prior to biopsy results and may influence the decision to initiate antiviral therapy. Furthermore, the early PCR detection can prompt earlier treatment when “gold standard” testing is not available as colonoscopy is relatively contraindicated in the presence of neutropenia. Our patient's CMV infection resolved without the use of antivirals, which we attribute to recovery of the immune system. However, it is possible that the use of early antiviral treatment could have led to shorter hospitalization and reduced complications.

Given the high morbidity and mortality associated with reactivated CMV disease, physicians may consider CMV testing, monitoring, and preemptive treatment in leukemic patients with high risk features who are undergoing chemotherapy. A high index of suspicion for CMV disease may be beneficial in those cancer patients who clinically deteriorate and may benefit from early antiviral therapy to improve prognosis.
